# Association of miR-196a2 and miR-27a polymorphisms with gestational diabetes mellitus susceptibility in a Chinese population

**DOI:** 10.3389/fendo.2023.1127336

**Published:** 2023-04-04

**Authors:** Qiaoli Zeng, Dehua Zou, Na Liu, Yue Wei, Jing Yang, Weibiao Wu, Fengqiong Han, Rongrong He, Runmin Guo

**Affiliations:** ^1^ Department of Internal Medicine, Shunde Women and Children’s Hospital (Maternity and Child Healthcare Hospital of Shunde Foshan), Guangdong Medical University, Foshan, Guangdong, China; ^2^ State Key Laboratory of Quality Research in Chinese Medicine, School of Pharmacy, Macau University of Science and Technology, Taipa, Macau, China; ^3^ Guangdong Engineering Research Center of Chinese Medicine & Disease Susceptibility, Jinan University, Guangzhou, Guangdong, China; ^4^ Key Laboratory of Research in Maternal and Child Medicine and Birth Defects, Guangdong Medical University, Foshan, Guangdong, China; ^5^ Matenal and Child Research Institute, Shunde Women and Children’s Hospital (Maternity and Child Healthcare Hospital of Shunde Foshan), Guangdong Medical University, Foshan, Guangdong, China; ^6^ Department of Pediatrics, Shunde Women and Children’s Hospital (Maternity and Child Healthcare Hospital of Shunde Foshan), Guangdong Medical University, Foshan, Guangdong, China; ^7^ Department of Ultrasound, Shunde Women and Children’s Hospital (Maternity and Child Healthcare Hospital of Shunde Foshan), Guangdong Medical University, Foshan, Guangdong, China; ^8^ Department of Ultrasound, Affiliated Hospital of Guangdong Medical University, Zhanjiang, Guangdong, China; ^9^ Department of Obstetric, Shunde Women and Children’s Hospital (Maternity and Child Healthcare Hospital of Shunde Foshan), Guangdong Medical University, Foshan, Guangdong, China; ^10^ Medical Genetics Laboratory, Shunde Women and Children’s Hospital (Maternity and Child Healthcare Hospital of Shunde Foshan), Guangdong Medical University, Foshan, Guangdong, China; ^11^ Department of endocrinology, Affiliated Hospital of Guangdong Medical University, Zhanjiang, Guangdong, China

**Keywords:** gestational diabetes mellitus (GDM), miR-196a2, miR-27a, rs11614913, rs895819, case-control study

## Abstract

**Introduction:**

MiR-196a2 and miR-27a play a key role in the regulation of the insulin signaling pathway. Previous studies have indicated that miR-27a rs895819 and miR-196a2 rs11614913 have a strong association with type 2 diabetes (T2DM), but very few studies have investigated their role in gestational diabetes mellitus (GDM).

**Methods:**

A total of 500 GDM patients and 502 control subjects were enrolled in this study. Using the SNPscan™ genotyping assay, rs11614913 and rs895819 were genotyped. In the data treatment process, the independent sample t test, logistic regression and chi-square test were used to evaluate the differences in genotype, allele, and haplotype distributions and their associations with GDM risk. One-way ANOVA was conducted to determine the differences in genotype and blood glucose level.

**Results:**

There were obvious differences in prepregnancy body mass index (pre-BMI), age, systolic blood pressure (SBP), diastolic blood pressure (DBP) and parity between GDM and healthy subjects (*P* < 0.05). After adjusting for the above factors, the miR-27a rs895819 C allele was still associated with an increased risk of GDM (C vs. T: OR=1.245; 95% CI: 1.011-1.533; *P* = 0.039) and the TT-CC genotype of rs11614913-rs895819 was related to an increased GDM risk (OR=3.989; 95% CI: 1.309-12.16; *P* = 0.015). In addition, the haplotype T-C had a positive interaction with GDM (OR=1.376; 95% CI: 1.075-1.790; *P*=0.018), especially in the 18.5 ≤ pre-BMI < 24 group (OR=1.403; 95% CI: 1.026-1.921; *P*=0.034). Moreover, the blood glucose level of the rs895819 CC genotype was significantly higher than that of the TT and TC genotypes (*P* < 0.05). The TT-CC genotype of rs11614913-rs895819 showed that the blood glucose level was significantly higher than that of the other genotypes.

**Discussion:**

Our findings suggest that miR-27a rs895819 is associated with increased GDM susceptibility and higher blood glucose levels.

## 1 Introduction

Gestational diabetes (GDM) is a common disease in pregnancy that is determined by the first diagnosis of hyperglycemia (1). GDM is harmful to the health of pregnant women and fetuses to a certain extent. For pregnant women, it may increase the incidence of complications, such as pregnancy hypertension, cardiovascular disease and glucose metabolism inhibition (2). For the fetus, there is a risk of premature birth and neonatal hypoglycemia (3). Therefore, to prevent and treat the occurrence of GDM, it is necessary to explore its pathogenesis and risk factors. The pathogenesis of GDM may include impaired insulin secretion and insulin resistance (2). Dietary, environmental and genetic factors contribute to GDM development (4), among which single nucleotide polymorphisms (SNPs) are an important genetic variation factor (5).

MicroRNAs (miRNAs) play a key regulatory role in the metabolic signaling pathway during pregnancy (6), which may influence islet β-cell differentiation and islet development (7). A growing number of studies have shown that SNPs in miRNAs have an impact on their maturation, expression and function. The dysregulation of miRNA expression is associated with cancer, diabetes and cardiovascular disease development (8, 9). It has been reported that miR-196a2 and miR-27a are involved in the regulation of the insulin signaling pathway and have a strong correlation with diabetes mellitus (DM) (10–20). There have been many previous studies on the association of miR-196a2 rs11614913 and miR-27a rs895819 polymorphisms with type 2 diabetes (T2DM) (21–29), but very few studies have investigated the association of these miRNAs with GDM (20).

The Oral Glucose Tolerance Test (OGTT) is currently regarded as the gold standard for diagnosing GDM (30), yet it is a cumbersome process, requiring fasting and multiple blood draws, and is associated with nausea and vomiting, resulting in reduced patient compliance. Additionally, the OGTT is performed between 24-28 weeks of gestation, providing a limited timeframe to implement interventions to improve pregnancy outcomes. Therefore, it is essential to find ways to increase patient compliance and facilitate early detection. In recent years, SNPs have been explored as potential molecular biomarkers for GDM screening (31). While the correlation between miR-27a rs895819, miR-196a2 rs11614913 and gestational diabetes has been less studied, the identification of sensitive and specific biomarkers through the detection of these SNPs may offer potential for GDM risk prediction and intervention strategies.

Therefore, this study evaluated the association between the single SNPs rs11614913 and rs895819, SNP-SNP and GDM risk and further explored the correlation between genotype and blood glucose level. We conducted a Chinese case-control study to assess whether miR-196a2 rs11614913 and miR-27a rs895819 are associated with GDM risk. Further meta-analysis was performed to estimate the relationships between rs11614913 and rs895819 and DM.

## 2 Materials and methods

### 2.1 Study subjects

This study protocol was approved by the Ethics Committee of Shunde Women and Children’s Hospital of Guangdong Medical University, and subjects for this study were selected through the following criteria: (i) voluntary informed consent; (ii) never diagnosed with diabetes; (iii) Han ethnicity; (iv) age not less than 18 years; (v) no pregnancy complications; and (vi) no glucose-lowering medication. A total of 1002 pregnant Chinese Han women were recruited, including 500 in the GDM group and 502 in the control group. Based on the GDM diagnostic criteria of the International Association of Diabetes and Pregnancy Study Groups (IADPSG), during 24-28 weeks of pregnancy, pregnant women took 75 g glucose for the glucose tolerance test (OGTT), and subjects with at least one glucose level measurement equal to or above the threshold value (fasting blood glucose level, FBP ≥ 5.1 mmol/L, 1 hour blood glucose level, 1 h-PG ≥ 10.0 mmol/L or 2 hour blood glucose level, 2 h-PG ≥ 8.5 mmol/L) were diagnosed with GDM, while subjects with normoglycemic levels were deemed healthy controls. This study was performed based on the principles of the Declaration of Helsinki.

### 2.2 Data collection

General clinical information, such as age, ethnicity, height, systolic blood pressure (SBP), diastolic blood pressure (DBP), prepregnancy weight, and parity (primipara or multipara) were gathered. The prepregnancy body mass index (pre-BMI, Kg/m2) was calculated as prepregnancy weight (Kg) divided by the square of the height (m^2^). According to BMI, the obesity criteria of Chinese people were divided into the following groups: obesity (≥28 Kg/m^2^), overweight (24 Kg/m^2^ ≤ BMI <28 Kg/m^2^), normal (18.5 Kg/m^2^ ≤ BMI <24 Kg/m^2^), and underweight (<18.5Kg/m^2^).

### 2.3 SNP genotyping

The QIAamp DNA blood kit (Qiagen, Germany) was used to extract genomic DNA. Genotypes of individual SNPs were detected using the SNPscan method, and the raw data were collected on an ABI3730XL sequencer and analyzed with GeneMapper 4.1 software (Applied Biosystems, USA) (Genesky Technologies Inc., Shanghai, China). The accuracy of genotyping results was ensured by further quality control.

### 2.4 Statistical analyses

All statistical analyses were performed using SPSS 20.0 software (SPSS, Chicago, IL, USA). Independent sample t test was used for comparison of continuous variables (mean ± standard deviation); discontinuous variables, including Hardy-Weinberg equilibrium (HWE) in the control group, were compared using chi-square tests. After adjusting for potential confounders (including age, pre-BMI, blood pressure, and parity), the association of SNP, SNP-SNP and risk of GDM was assessed by dominance ratio (OR) and 95% confidence interval (CI) using binary logistic regression analysis. One-way ANOVA was used to analyze the correlation between SNP, SNP-SNP and blood glucose levels. The least significant difference (LSD) method was used for multiple comparisons. Bilateral *P* < 0.05 was statistically significant.

### 2.5 Bioinformatics analyses

Utilized the UCSC database (http://genome.ucsc.edu/) to locate SNPs with a minimum allele frequency of one percent or higher. The effect of variation on RNA folding and the stability of mRNA secondary structure was analyzed using RNAfold Web Servers (http://rna.tbi.univie.ac.at/cgi-bin/RNAWebSuite/RNAfold.cgi). In addition, the miRWalk (http://mirwalk.umm.uni-heidelberg.de/) online tool was used to predict the GDM-related genes that were coregulated by miR-196a2 and miR-27a.

### 2.6 Meta-analysis

Different combinations of the terms rs11614913, rs895819, gestational diabetes mellitus, GDM, type 2 diabetes mellitus, T2DM and type 1 diabetes mellitus, T1DM were used to comprehensively search the literature through the PubMed, Chinese National Knowledge Infrastructure and Google Scholar databases with no limitations. The inclusion criteria were case-control or cohort studies that assessed the association of rs11614913 and rs895819 with GDM/T2DM/T1DM with sufficient raw data. Studies that did not meet the diagnostic criteria and studies with data that were not in HWE were excluded. Two authors supervised each other to extract the basic data in the article. The overall and subgroup meta-analysis of five genetic models used the fixed or random effects model according to the level of heterogeneity (32). Publication bias was determined using Egger’s and Begg’s tests. All meta-analyses were performed using STATA v.16.0 software (Stata Corporation, TX, USA).

## 3 Results

### 3.1 General clinical characteristics of the subjects

This case-control study included 500 GDM and 502 healthy controls for whom the genotypes of miR-27a rs895819 and miR-196a2 rs11614913 were detected. Clinical baseline information is listed in **Table 1**. The mean age, pre-BMI, SBP, DBP, and blood glucose levels were significantly higher in the GDM group than in the control group *(P* < 0.05). Moreover, the parity of the GDM group was significantly different from that of the control group (*P* < 0.05).

**Table 1:** Basic and stratified characteristic of participants of the study.

Variables	Cases (%)	Controls (%)	t/x2	*P*
Age, year (mean ± SD)	31.01 ± 4.32	28.66 ± 4.37	-8.56	**<0.001**
			49.2	**<0.001**
<30	192 (38.4)	304 (60.6)		
≥30	308 (61.6)	198 (39.4)		
pre-BMI, kg/m2	21.51 ± 3.10	20.53 ± 2.58	-5.42	**<0.001**
			27.8	**<0.001**
<18.5	67 (13.4)	95 (18.9)		
18.5 ≤ BMI < 24	336 (67.2)	365 (72.7)		
≥24	97 (19.4)	42 (8.3)		
SBP, mmHg	116.69 ± 10.96	114.33 ± 10.18	-3.53	**<0.001**
DBP, mmHg	69.77 ± 7.80	68.23 ± 7.26	-3.23	**0.001**
FBP, mmol/L	4.82 ± 0.64	4.50 ± 0.31	-9.75	**<0.001**
1h-PG, mmol/L	10.17 ± 1.60	7.66 ± 1.27	-26.22	**<0.001**
2h-PG, mmol/L	8.91 ± 1.60	6.69 ± 0.99	-25.85	**<0.001**
Parity (n)			8.88	**0.003**
Primipara	210 (42)	258 (51.4)		
Multipara	290 (58)	244 (48.6)		

pre-BMI, pre-gestational body mass index; SBP, systolic blood pressure; DBP, diastolic blood pressure; FBP, fasting blood glucose level; 1h-PG, 1 hour blood glucose level; 2h-PG, 2 hour blood glucose level.

### 3.2 The association of rs11614913 and rs895819 with GDM risk

#### 3.2.1 Overall analysis results

**Table 2** shows the results of Hardy-Weinberg equilibrium (HWE) analysis and minor allele frequencies (MAF) for the 2 SNPs in the control group. The results were consistent with HWE (*P* > 0.05). The (unadjusted and adjusted) OR and 95% CI of the correlation between genotype and GDM were estimated in five models (codominant homozygous, codominant heterozygous, dominant, recessive and allele models) for each polymorphism. Before adjustment, the results showed the rs895819 dominant model (CC+TC vs. TT: OR=1.293; 95% CI: 1.008-1.658; *P* = 0.043) and the rs895819 allele model (C vs. T: OR=1.257; 95% CI: 1.032-1.532; *P* = 0.023) associated with increased GDM risk. After adjusting for age, pre-BMI, SBP, DBP, and parity, the results of the rs895819 allele model (C vs. T: OR=1.245; 95% CI: 1.011-1.533; *P* = 0.039) remained significantly associated with increased GDM risk (**Table 3**). However, no significant correlation with GDM risk was found for rs11614913 (**Table 3**).

**Table 2:** SNPs information and HWE test in the controls.

SNP	Min/Maj	Chr. position	MAF	HWE(P)
rs11614913	C/T	chr12:53991815	0.462	0.411
rs895819	C/T	chr19:13836478	0.247	0.996

HWE, Hardy–Weinberg equilibrium; Min, minor allele; Maj, major allele; MAF, frequency of minor allele.

**Table 3:** The associations between SNPs and GDM risk in overall subjects.

SNP	Genetic Models	Cases (freq) (n=500)	Controls (freq) (n=502)	Crude OR (95 % CI)	Crude *P*	Adjusted OR(95 % CI)	Adjusted *P*
rs895819	Codominant model						
	TT	252 (0.504)	285 (0.567)	1(ref)		1(ref)	
	TC	204 (0.408)	186 (0.37)	1.240 (0.955-1.611)	0.106	1.233 (0.936-1.622)	0.136
	CC	44 (0.088)	31 (0.061)	1.605 (0.984-2.620)	0.058	1.576 (0.939-2.644)	0.085
	Aelle model						
	T	708 (0.708)	756 (0.752)	1(ref)		1(ref)	
	C	292 (0.292)	248 (0.247)	1.257 (1.032-1.532)	**0.023**	1.245 (1.011-1.533)	**0.039**
	Dominant Model						
	TT	252 (0.504)	285 (0.567)	1(ref)		1(ref)	
	CC+TC	248 (0.496)	217 (0.433)	1.293 (1.008-1.658)	**0.043**	1.281 (0.985-1.665)	0.064
	Recessive Model						
	TC+TT	456 (0.912)	471 (0.939)	1(ref)		1(ref)	
	CC	44 (0.088)	31 (0.061)	1.466 (0.910-2.363)	0.116	1.440 (0.870-2.384)	0.156
rs11614913	Codominant model						
	TT	142 (0.284)	148 (0.294)	1(ref)		1(ref)	
	TC	254 (0.508)	245 (0.488)	1.081 (0.809-1.443)	0.6	1.003 (0.738-1.362)	0.985
	CC	104 (0.208)	109 (0.217)	0.994 (0.698-1.417)	0.975	0.930 (0.639-1.353)	0.704
	Aelle model						
	T	538 (0.538)	541 (0.538)	1(ref)		1(ref)	
	C	462 (0.462)	463 (0.462)	1.003 (0.842-1.196)	0.97	0.968 (0.804-1.165)	0.728
	Dominant Model						
	TT	142 (0.284)	148 (0.294)	1(ref)		1(ref)	
	CC+TC	358 (0.716)	354 (0.706)	1.054 (0.802-1.385)	0.706	0.981 (0.734-1.309)	0.894
	Recessive Model						
	TC+TT	396 (0.792)	393 (0.783)	1(ref)		1(ref)	
	CC	104 (0.208)	109 (0.217)	0.947 (0.700-1.282)	0.724	0.928 (0.673-1.279)	0.648

Adjusted P value calculated by logistic regression with adjustment for age, pre-BMI, SBP,DBP and parity.

#### 3.2.2 Stratified analysis results

Subsequently, the association of the 2 SNPs in 5 models with GDM susceptibility was tested using stratified analysis by age or pre-BMI. Notably, for the rs895819 dominant model (CC+TC vs. TT: OR=1.515; 95% CI: 1.053-2.179; *P* = 0.025), rs895819 codominant heterozygote model (TC vs. TT: OR=1.514; 95% CI: 1.036-2.214; *P* = 0.032) and rs895819 allele model (C vs. T: OR=1.353; 95% CI: 1.015-1.802; *P* = 0.039) the results showed a significantly increased GDM risk in subjects younger than 30 years of age. In the 18.5 ≤ pre-BMI <24 group, the results of the rs895819 dominant model (CC+TC vs. TT: OR=1.434; 95% CI: 1.064-1.933; *P* = 0.018), rs895819 codominant heterozygote model (TC vs. TT: OR=1.402; 95% CI: 1.024-1.918; *P* = 0.035) and rs895819 allele model (C vs. T: OR=1.335; 95% CI: 1.052-1.693; *P* = 0.017) showed that rs895819 was significantly related to increased GDM risk; however, after correction, no significant difference was found. In addition, no significant correlation with GDM risk was found for rs11614913 (**Supplementary Table 1**-**4**).

### 3.3 The association between rs11614913-rs895819 and GDM risk

We further investigated the effect of rs11614913-rs895819 interactions. The model included three genotypes and alleles of miRNA polymorphisms. The results after adjusting for age, pre-BMI, SBP, DBP, and parity showed that the TT-CC genotype of miR-196a2 rs11614913 and miR-27a rs895819 was associated with increased GDM risk (OR=3.989; 95% CI: 1.309-12.16; *P* = 0.015). In addition, the haplotype T-C was significantly associated with increased GDM risk (OR=1.376; 95% CI: 1.075-1.790; *P* = 0.018) (**Table 4**), especially in the group with 18.5 ≤ pre-BMI < 24 (OR=1.403; 95% CI: 1.026-1.921; *P* = 0.034) (**Supplementary Tables 5**, **6**).

**Table 4:** The associations between combined genotype/aelle and GDM risk.

Genotype combination		Cases (freq)	Controls (freq)	Crude OR(95 % CI)	Crude *P*	Adjusted OR(95 % CI)	Adjusted *P*
rs11614913	rs895819	(n=500)	(n=502)				
TT	TT	65 (0.13)	85(0.169)	1(ref)		1(ref)	
	TC	63(0.126)	58(0.116)	1.420 (0.878-2.298)	0.153	1.414 (0.852-2.347)	0.18
	CC	14(0.028)	5(0.010)	3.661 (1.255-10.69)	**0.018**	3.989 (1.309-12.16)	**0.015**
TC	TT	136(0.272)	139(0.277)	1.279 (0.857-1.909)	0.227	1.165 (0.762-1.781)	0.482
	TC	97(0.194)	85(0.169)	1.492 (0.966-2.305)	0.071	1.455 (0.917-2.307)	0.111
	CC	21(0.042)	21(0.041)	1.307 (0.659-2.596)	0.443	1.268 (0.613-2.622)	0.523
CC	TT	51(0.102)	61(0.121)	1.093 (0.668-1.789)	0.723	1.140 (0.675-1.925)	0.625
	TC	44(0.088)	43(0.085)	1.338 (0.787-2.273)	0.281	1.159 (0.663-2.029)	0.604
	CC	9(0.018)	5(0.010)	2.354 (0.753-7.359)	0.141	1.654 (0.488-5.604)	0.419
**Aelle combination**		Cases (freq)	Controls (freq)	Crude OR(95 % CI)	Crude *P*	Adjusted OR(95 % CI)	Adjusted *P*
**rs11614913**	**rs895819**	(2n=1000)	(2n=1004)				
T	T	329(0.329)	367(0.366)	1(ref)		1(ref)	
	C	209(0.209)	174(0.173)	1.340 (1.043-1.721)	**0.022**	1.376 (1.057-1.790)	**0.018**
C	T	379(0.379)	389(0.387)	1.087 (0.885-1.335)	0.427	1.078 (0.868-1.339)	0.495
	C	83(0.083)	74(0.074)	1.251 (0.884-1.770)	0.205	1.116 (0.773-1.610)	0.559

Adjusted P value calculated by logistic regression with adjustment for age, pre-BMI, SBP, DBP and parity.

### 3.4 Association between genotype and blood glucose level

The results of the OGTT experiment showed that the 2-hour blood glucose level of the CC genotype of rs895819 was significantly higher than those of the TT and TC genotypes (*P* < 0.05)(**Table 5**). For the interaction genotype of rs11614913-rs895819, the fasting blood glucose level of the CC-TC genotype was higher than that of TC-TT (*P* < 0.05), and the 1-hour and 2-hour blood glucose levels of the TT-CC genotype were significantly higher than those of the TT-TC, TC-TC and CC-TT genotypes (*P* < 0.05) (**Table 6**).

**Table 5:** Relationship between polymorphisms genotype and blood glucose levels.

SNP	Genotype	FBG (mmol/L)	1 h-PG (mmol/L)	2 h-PG (mmol/L)
**rs11614913**	TT	4.650 ± 0.402	8.919 ± 1.734	7.822 ± 1.552
	TC	4.661 ± 0.426	9.036 ± 1.893	7.927 ± 1.718
	CC	4.714 ± 0.840	9.031 ± 2.189	7.833 ± 2.023
	F	0.912	0.348	0.385
	*P*	>0.05** **	>0.05	>0.05
**rs895819**	TT	4.641 ± 0.440	8.947 ± 1.890	7.764 ± 1.687^a^
	TC	4.704 ± 0.666	9.004 ± 1.998	7.917 ± 1.788^b^
	CC	4.690 ± 0.375	9.387 ± 1.630	8.483 ± 1.791
	F	1.505	1.581	5.31
	*P*	>0.05** **	>0.05	**<0.05 **

a LSD was used to compare the blood glucose levels of three rs895819 genotypes: the difference of 2-hour blood glucose between CC and TT genotypes was statistically significant, P = 0.001.

b LSD was used to compare the blood glucose levels of three rs895819 genotypes: the difference of 2-hour blood glucose between CC and TC genotypes was statistically significant, P = 0.014.

**Table 6:** Relationship between polymorphisms combined genotype and blood glucose levels.

Genotype combination		FBG(mmol/L)	1 h-PG(mmol/L)	2 h-PG(mmol/L)
rs11614913	rs895819			
TT	TT	4.632 ± 0.408	8.733 ± 1.773^b^	7.583 ± 1.538^cd ^
	TC	4.678 ± 0.405	8.972 ± 1.654^b^	7.924 ± 1.480^c^
	CC	4.618 ± 0.338	9.992 ± 1.598	8.985 ± 1.586
TC	TT	4.638 ± 0.442^a^	9.092 ± 1.851	7.896 ± 1.715^c^
	TC	4.687 ± 0.421	8.951 ± 1.998^b^	7.928 ± 1.732^c^
	CC	4.698 ± 0.335	9.046 ± 1.706	8.135 ± 1.699
CC	TT	4.660 ± 0.482	8.871 ± 2.106^b^	7.675 ± 1.788^cd^
	TC	4.772 ± 1.170	9.157 ± 2.398	7.886 ± 2.245^c ^
	CC	4.770 ± 0.522	9.522 ± 1.249	8.781 ± 2.200
	F	0.697	1.276	2.159
	*P*	>0.05** **	>0.05	**＜0.05 **

^a^ LSD was used to compare the blood glucose levels of nine genotype combinations: the difference of FBG between CC-TC and TC-TT genotype combination was statistically significant, P < 0.05.

^b^ LSD was used to compare the blood glucose levels of nine genotype combinations: the difference of 1-hour blood glucose between TT-CC and other genotype combination were statistically significant, P < 0.05.

^c^ LSD was used to compare the blood glucose levels of nine genotype combinations: the difference of 2-hour blood glucose between TT-CC and other genotype combination were statistically significant, P < 0.05.

^d^ LSD was used to compare the blood glucose levels of nine genotype combinations: the difference of 2-hour blood glucose between CC-CC and other genotype combination were statistically significant, P < 0.05.

### 3.5 Effects of variants on miRNA secondary structure

**Figure 1:**
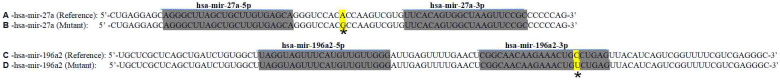
RNA precursor sequence and mutation sites (marked with asterisk). A hsa-miR-27a (reference), B hsa-miR-27a (mutant), C hsa-miR-196a2 (reference), D hsa-miR-196a2 (mutant).

**Figure 2:**
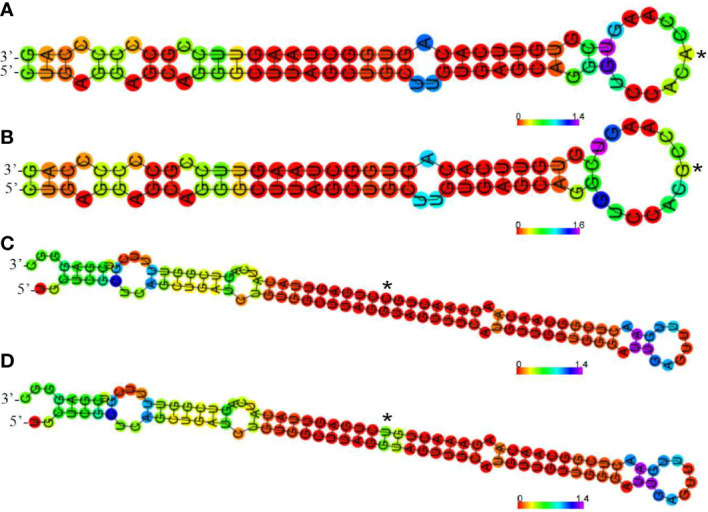
Centroid secondary structure of pre-mir-27a and pre-mir-196a2. The size of the miRNA hairpin loop increases when the rs895819 T allele is replaced by the C allele. **(A)** hsa-miR-27a (reference), **(B)** hsa-miR-27a (mutant), **(C)** hsa-miR-196a2 (reference), **(D)** hsa-miR-196a2 (mutant).

**Figure 3:**
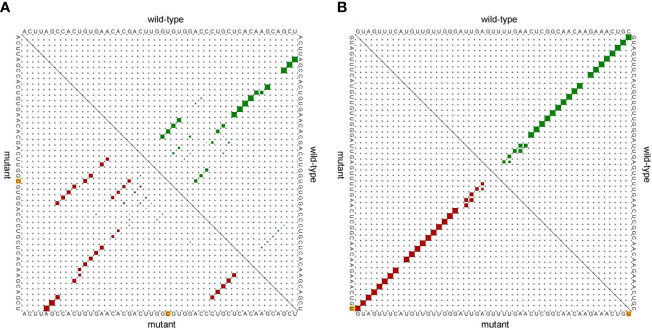
Base pair probability of local region. **(A)** miR-27a rs895819 T>C, **(B)** miR-196a2 rs11614913 C>T.

The locations of the SNP mutation sites in the studied miRNAs are shown in **Figure 1**. The analysis of the effect of SNPs on the local miRNA structure showed that the centroid secondary structure in dot-bracket notation with a minimum free energy (MFE) of the T and C rs895819 alleles are -34.3 kcal/mol and -30.4 kcal/mol, respectively, and the size of the miRNA hairpin loop increases when the T allele is replaced by the C allele. (**Figures 2A, B**). Thermodynamically, the lower the MFE, the more stable the miRNA structure. Thus, these variations may affect the processing of pre-miRNAs.The centroid secondary structure in dot-bracket notation with an MFE of the C and T rs11614913 alleles are −49.9 kcal/mol and -44.3 kcal/mol, respectively (**Figures 2C, D**). This suggests that the local miRNA structure of the C allele may be more stable than that of the T allele. **Figure 3** shows the base pair probabilities of wild-type and mutant-type, suggesting the difference between wild-type and mutant-type.

### 3.6 Meta-analysis results

**Figure 4:**
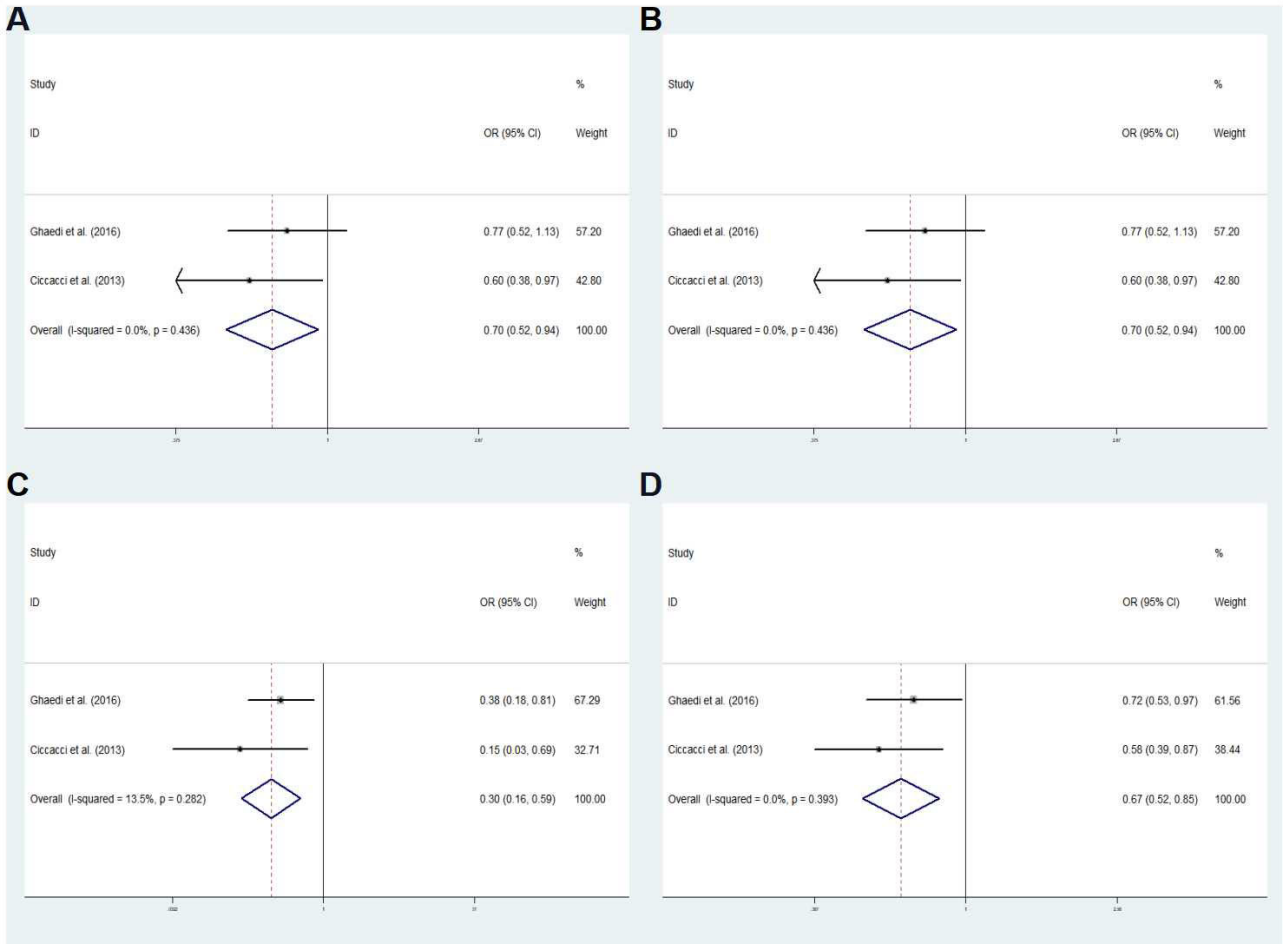
Subgroup meta-analysis for the association between miR-27a rs895819 and T2DM susceptibility in a Caucasian population in fixed effects model. **(A)** Dominant model, CC+TC vs. TT. **(B)** Recessive model, CC vs. TC+TT. **(C)** co-dominant homozygous model, CC vs. TT. **(D)** Allele model C vs. T. OR odds ratio, CI, confidence interval; I2: measurement to quantify the degree of heterogeneity in meta-analyses.

The final analysis included 12 studies (including our study): 6 studies related to rs11614913 and GDM/T2DM/T1DM (1/4/1) and 7 studies related to rs895819 and GDM/T2DM (2/5). **Table 7** shows the characteristics of the studies. In the overall analysis, no significant associations were found between rs11614913 and rs895819 and DM. In the subgroup meta-analysis, the results of the rs895819 dominant model (CC+TC vs. TT: OR=0.699; 95% CI: 0.518-0.943; *P* = 0.019), rs895819 recessive model (CC vs. TC+TT: OR=0.365; 95% CI: 0.190-0.701; *P* = 0.002), rs895819 codominant homozygous model (CC vs. TT: OR=0.305; 95% CI: 0.156-0.595; *P <*0.001) and rs895819 allele model (C vs. T: OR=0.667; 95% CI: 0.524-0.848; *P* = 0.001) showed that the tested models were associated with decreased T2DM risk in a Caucasian population (**Figure 4**). No significant difference was found in other groups (data not shown).

**Table 7:** Characteristics of each study included in the meta-analysis.

SNP							Allele distribution				Genotype distribution						
							Cases (n)		Controls (n)		Cases (n)			Controls (n)			
rs11614913	Athor	Year	Ethnicity	Type	Cases (n)	Controls(n)	C	T	C	T	CC	CT	TT	CC	CT	TT	HWE
	Zeng et al.(Our study)	2023	Asian	GDM	500	502	462	538	463	541	104	254	142	109	245	148	>0.05
	MIR et al.	2022	Caucasian	T2DM	100	100	145	55	165	35	51	43	6	70	25	5	>0.05
	Khan et al.	2021	Caucasian	T2DM	338	236	346	330	333	139	84	178	76	130	73	33	>0.05
	Huang et al.	2021	Asian	T2DM	497	782	413	581	691	873	81	251	165	138	415	229	>0.05
	Ibrahim et al.	2019	Caucasian	T1DM	150	150	175	125	206	94	59	57	34	71	64	15	>0.05
	Buraczynska et al.	2014	Caucasian	T2DM	920	834	1224	616	1001	667	414	396	110	292	417	125	>0.05
rs895819	Athor	Year	Ethnicity	Type	Cases (n)	Controls(n)	T	C	T	C	TT	TC	CC	TT	TC	CC	HWE
	Zeng et al. (Our study)	2023	Asian	GDM	500	502	708	292	756	248	252	204	44	285	186	31	>0.05
	Choi et al.	2022	Asian	T2DM	238	247	317	159	277	217	106	105	27	84	109	54	>0.05
	Ghaedi et al.	2016	Caucasian	T2DM	204	209	301	107	280	138	108	85	11	97	86	26	>0.05
	Wang et al.	2015	Asian	T2DM	995	967	1469	521	1415	519	554	361	80	526	363	78	>0.05
	Li et al.	2015	Asian	T2DM	738	610	1064	412	900	320	371	322	45	330	240	40	>0.05
	Wang et al.	2014	Asian	GDM	837	848	1293	381	1257	439	482	329	26	469	319	60	>0.05
	Ciccacci et al.	2013	Caucasian	T2DM	148	147	247	49	219	75	101	45	2	83	53	11	>0.05

n number, T1DM type 1 diabetes mellitus, T2DM type 2 diabetes mellitus, GDM gestational diabetes mellitus, HWE Hardy–Weinberg equilibrium.

## 4 Discussion

MiRNAs affect gene expression through posttranscriptional regulation and are involved in many important physiological processes (21). Polymorphisms of miRNAs may affect their maturation, expression and function, which may lead to human disease susceptibility (22). It has been found that miRNA polymorphisms are associated with a variety of cancers, T2DM, GDM and cardiovascular diseases (23–25). In this study, we evaluated the associations between miR-27a rs895819 and miR-196a2 rs11614913 and GDM susceptibility in a Chinese population.

The results showed that the miR-27a rs895819 C allele was associated with increased GDM risk, and a previous study indicated that the miR-27a CC genotype was associated with increased T2DM risk in an overweight Chinese population (18). Zhu et al. showed that miR-27a rs895819 variant genotypes were also associated with an increased risk of T2MD in both the age ≥ 60 years (GG genotype) and male subgroup (AG genotype and dominant model) (26). MiRNA SNPs may contribute to the development of GDM by changing the expression of target genes. The results of this study showed that the rs895819 mutant C allele increased the production of mature miR-27a and suppressed the expression of its target genes compared to the wild T allele (27). Furthermore, human Drosha selectively cleaves RNA hairpins with larger terminal loops (28). Thus, as long as the size of the miRNA loop is altered by mutation or deletion, the maturation process of Drosha is affected. The miR-27a secondary structure analysis found that the size of the miRNA hairpin loop increases when the T allele is replaced by the C allele. This enlargement has been shown to accelerate the maturation of miR-27a, resulting in the upregulation of miR-27a (28). Interestingly, the expression of the miR-27a rs895819 AG and GG genotypes was significantly higher than that of the AA genotype (29). The *peroxisome proliferator-activated receptorγ*(*PPARγ*) is the target gene of miR-27a, and miR-27a rs895819 variants may further negatively regulate the expression of the *PPARγ* gene, it may directly down-regulates the adiponectin (33). Adiponectin deficiency is strongly associated with insulin resistance in pregnancy, Khosrowbeygi et al. showed that the adiponectin level of the GDM group was significantly lower than that of healthy pregnant women (34). Thus miR-27a rs895819 variants is considered to be associated with insulin resistance and diabetes.

However, the results of studies in Caucasians were in contrast to these results. Ciccacci et al. showed that the miR-27a rs895819 G allele played a protective role against T2DM in an Italian study (17), and Ghaedi et al. also found that the miR-27a rs895819 C allele played a protective role against T2DM in an Iranian cohort (16). Our meta-analysis verified the above results. These conflicting results with those of our study may be related to ethnic differences in the study populations. In a recent study of a Korean population, it was found that the G allele in a recessive model and the GG genotype of miR-27a rs895819 were significantly associated with decreased T2DM risk, but the sample sizes of T2DM and healthy controls were only 238 and 247, respectively (15). The results of only one study on GDM showed that the miR-27a rs895819 C allele decreased GDM risk in a Chinese population (20), which is contrary to our results. After summarizing the data of our study and the above study, we conducted a meta-analysis and found no correlation between rs895819 and GDM. Therefore, more research on GDM is especially important.

Moreover, miR-196a2 may regulate the insulin signaling pathway, and miR-196a2 variants are involved in T2DM development (10, 35). Rs11614913 is located in the 3p arm of miR-196a2 (36), which may affect the maturation of pre-miRNAs and target gene binding (37). Previous studies showed that the miR−196a2 rs11614913 T allele and CT genotype were associated with an increased T2DM risk in the Saudi Arabian population. Huang et al. showed that the rs11614913 C allele was significantly associated with decreased T2DM susceptibility in the smoking subgroup (13), but a study of a Pakistani population found an increased association between the miR−196a2 rs11614913 C allele and T2DM risk. Our results did not find a significant correlation between miR-196a2 rs11614913 and GDM in the Chinese population. The meta-analysis results did not find a significant association between miR-196a2 rs11614913 and DM risk. These contradicting findings may be related to the different sample sizes of studies and ethnic differences.

The combined SNP genotype analysis indicated that the TT-CC genotype of miR-196a2 rs11614913 and miR-27a rs895819 was associated with an increased risk of GDM susceptibility. This detection of the interaction of rs11614913 and rs895819 in GDM was defined as an epistatic influence, which generally explains the absence or underestimation of heritability when only a single SNP is included in a disease susceptibility study (38). According to the results of our research, miR-196a2 rs11614913 probably has no impact on GDM. However, miR-196a2 rs11614913 and miR-27a rs895819 may jointly affect the development of GDM. Remarkably, the combined genotype and haplotype methods have high potential for application in association research (39). In the haplotype results, the allele combination T-C haplotype of miR-196a2 rs11614913 and miR-27a rs895819 was significantly associated with increased GDM risk, especially in the group with 18.5 ≤ pre-BMI <24. The results of the miRWalk database analysis showed that both miR-196a2 and miR-27a can target the Adiponectin gene, which is related to GDM. MiR-196a2 rs11614913 and miR-27a rs895819 variants may negatively regulate Adiponectin gene expression and increase susceptibility to GDM. Therefore, further functional verification is necessary.

The results of correlation analysis between genotype and blood glucose level showed that the 2-h blood glucose level of the miR-27a rs895819 CC genotype was significantly higher than that of the TT and TC genotypes. The 1-h and 2-h blood glucose levels of the TT-CC genotypes of rs11614913 and rs895819 were significantly higher than those of other combinations. Previous studies have shown that miR-27a in cluster C was positively correlated with fasting blood glucose level, which may play a key role in early hyperglycemia and contribute to the development of diabetes (40).

## 5 Conclusions

In general, our research is the first to confirm that miR-27a rs895819 may contribute to GDM susceptibility in pregnant Chinese women. However, one of the limitations of this study is the limited sample size. In addition, multicenter and further functional studies are needed to gain more insight into the association between rs895819 and GDM. Importantly, future research should verify some selected targets through luciferase analysis and evaluate the regulatory effect of these miRNA mutations on target gene expression.

## Data availability statement

The original contributions presented in the study are included in the article/**Supplementary Material**. Further inquiries can be directed to the corresponding author.

## Ethics statement

The study was agreed by the Ethics Committee of Shunde Women and Children’s Hospital of Guangdong Medical University (Maternity and Child Healthcare Hospital of Shunde Foshan). The patients/participants provided their written informed consent to participate in this study.

## Author contributions

QZ, DZ and NL contributed equally to this study. QZ, JY, WW and FH collected clinical data and samples, QZ, DZ and NL did data analyzes, QZ, DZ, YW and RG wrote the manuscript. FH, RH and RG supervised the whole research. All authors contributed to the article and approved the submitted version.
